# Greenlandic patients with colorectal cancer: symptomatology, primary investigations and differences in diagnostic intervals between Nuuk and the rest of the country

**DOI:** 10.1080/22423982.2017.1344086

**Published:** 2017-07-14

**Authors:** Johan Tolstrup, Rasmus Chemnitz Madsen, Maria Vandborg Sneftrup, Birgit Niclasen

**Affiliations:** ^a^ Surgical Department, Dronning Ingrids Hospital, Nuuk, Greenland; ^b^ Department of public Health, Aarhus University, Aarhus, Denmark; ^c^ Department of Health, Nuuk, Greenland

**Keywords:** Colorectal cancer, Greenland, Primary care, practitioners’ delay, hospital delay, symptomatology, diagnostic interval

## Abstract

**Background**: Colorectal cancer (CRC) is a potentially fatal disease, and expedited referral and treatment is needed to ensure early detection.

**Objective**: We aimed to assess the symptomatology of Greenlandic patients with CRC and the primary investigations initiated before referral to Dronning Ingrids Hospital in Nuuk for further diagnostic workup. Primary care interval (first consultation until referral), hospital interval (referral until diagnosis) and diagnostic interval (first consultation until diagnosis) were calculated and compared between patients living in Nuuk and in other places in Greenland (“the Coast”).

**Design**: This was a retrospective, register-based study of all patients in Greenland diagnosed with CRC from 2008 through 2011. Medical history was obtained and investigated by reviewing the primary care charts.

**Results**: In total 113 patients were identified from the Greenlandic cancer database or pathology reports. About 80% of the patients were asked about blood in the stools and changes of bowel habits, and the majority responded positively to this. Abdominal examination was performed for 78%, 65% had a rectal examination performed, 22% a proctoscopy performed and 51% a haemoglobin level measured.

The median primary care interval was 4 days in Nuuk vs. 55 days for patients from “the Coast” (p=0.01); the median diagnostic interval was 55 days in Nuuk vs. 95 days for patients from “the Coast” (p=0.04). Median hospital interval was similar for both groups (23 days vs 24 days; p=0.86). Women had a median primary care interval of 70 days vs. 15 days for men (p=0.06).

**Conclusions**: Patients with CRC presented classic symptomatology of CRC. Primary care interval and diagnostic interval were significantly longer for patients from “the Coast” compared with Nuuk. Women tended to have longer primary care interval. A more standardised examination should be implemented and a national CRC screening programme should be considered to reduce the difference in diagnostic interval and ensure timely referral.

## Introduction

Colorectal cancer (CRC) is the second most commonly diagnosed cancer in Greenland, only surpassed by lung cancer, and it is a frequent cause of morbidity and mortality [1]. The cost effectiveness of a national population screening programme for CRC is under investigation, but at present only patients with a family history are screened and only patients suspected for CRC are referred to further diagnostics. Two of the major aims of the national plan against cancer are early diagnosis and to strengthen the collaboration around the patient between different parts of the health care system. Regarding CRC, the national plan against cancer recommends closer surveillance of incidence, treatment and survival [2].

In Greenland the epidemiological aspects of CRC are not well described, but a cohort study from 1973 to 1997 showed an increase in CRC comparable with the incidence seen in Denmark at that time [3]. However, in a study including data from 2000 to 2009 [4] a slightly lower incidence rate of CRC was found in Greenland, compared with Denmark. We found no valid data on CRC mortality rates in Greenland.

Greenland has 56,000 inhabitants living in 16 towns and about 60 settlements. Providing health care is a governmental obligation, and all health care is free of charge. The first point of contact for the patient with CRC is usually a primary care setting. According to their symptoms, further local actions may be initiated or, if the suspicion of cancer is strong, the patient can be referred to the national hospital in Greenland (Dronning Ingrids Hospital (DIH), Nuuk), usually for colonoscopy. In Greenland Danish guidelines regarding referral are used, and according to these patients above 40 years of age with the following symptoms of CRC should be referred to endoscopy [5]: blood in the stools, changes of bowel habits for more than 4 weeks, bleeding anaemia or significant unspecific symptoms such as weight loss and abdominal pain. The referring physician or GP should address the abovementioned symptoms and determine whether the patient has a high or average risk of CRC based on symptoms or on family history. A clinical examination should be performed, including abdominal examination, rectal examination and gynaecological examination, and haemoglobin should be measured to identify a potential iron deficiency anaemia (IDA) [5].

Only approximately a quarter of the population in Greenland live in Nuuk where the national hospital (DIH) is situated. Therefore many patients who may suffer from CRC need to travel hundreds of kilometres from the “the Coast” to be properly diagnosed. Furthermore, the small hospitals and health care clinics in these areas are challenged by a high turnover of medical staff (physicians, nurses etc.), which may compromise the quality of care.

The diagnostic interval depends on the “primary care interval”, which is time from first consultation in primary care until date of referral (formerly called “practitioner delay”), and “hospital interval”, which is time from referral until diagnosis (formerly called “hospital delay”) [6]. Although CRC develops slowly, and no certain association between duration of cancer symptoms and stage (a prognostic marker) has been proven, a prolonged diagnostic interval may result in risk of perforation or obstruction of the colon and a more stressful and inconvenient course of disease for the patient [7,8]. Some evidence also suggests that a longer diagnostic interval may increase mortality for patients with CRC [9]. Also, there is strong evidence to support that early detection of CRC reduces mortality [10].

Due to logistics as well as the local opportunities for early diagnosis it was hypothesised that patients with CRC living outside Nuuk had a longer diagnostic interval than patients from Nuuk. To assess this, we wished to estimate the primary care interval and the hospital interval for Greenlandic patients suffering from CRC. Finally, we wished to explore the symptomatology of the patients with CRC and to see if the patients had been properly questioned and examined before referral to Nuuk.

## Material and methods

Ethical approval was granted by the scientific ethical committee of Greenland. All patients living in Greenland and diagnosed with CRC by histological examination from 2008 through 2011 were included in this study. The National Greenlandic Cancer Register was searched for patients with CRC in the period 2008 through 2011. To find all cases, all pathology reports obtained at the Surgical Department at DIH in the same period were investigated. This was done because earlier research in Greenland has shown that not all patients with cancer are properly reported to the Greenlandic Cancer Register [2]. Initially, patients diagnosed in 2007 were included, but since pathology reports had been destroyed for this year, these patients were excluded. In accordance with the pathological definition of CRC, we included patients with the following types of cancer: adenocarcinomas of glandular type, low differentiated adenocarcinomas, undifferentiated carcinomas, medullary carcinomas, signet ring cell carcinomas, adenosquamous carcinomas and mucinous adenocarcinomas.

Patients were excluded if they had a prior diagnosis of CRC and if the pathology findings were different from the histological characteristics stated above. This was done by reading through the medical records of the patients and by consulting the Danish pathology register (Patobank) where all pathology reports from both Denmark and Greenland are registered. Basic descriptive data were obtained on each patient. Valid data regarding co-morbidity were not available.

### Database of symptoms

Medical files from the local treatment facilities were obtained electronically from “Aeskulap”, which is the medical chart where the consultation is documented as well as the exact date of the meeting. A database with relevant symptoms of CRC was created. All medical files from physicians, nurses and other health workers were reviewed to identify and register symptoms of CRC.

The following symptoms were registered: blood in the stools (described as either red or black), changes of bowel habits, weight loss, general malaise (nausea, vomiting and fatigue), abdominal pain, diarrhoea, constipation, problems with emptying the bowel. We also noted what symptoms the patients presented with. Initially symptoms of anaemia were also registered (dizziness, fatigue and palpitations), but these symptoms could not be separated from “general malaise”, thus we chose to present these categories together. The final definition of general malaise/anaemia was: nausea, vomiting, fatigue, dizziness and palpitations. The following clinical examinations that were carried out prior to referral to DIH were registered: abdominal examination, rectal examination, gynaecological examination and proctoscopy. We noted under what suspicion the patient was referred. Finally, it was noted if the haemoglobin level had been measured and if anaemia was present (defined as haemoglobin <7mmol/L for women and haemoglobin <8mmol/L for men). Three time intervals were calculated: number of days from the first consultation in primary care to the date of referral to DIH (primary care interval), number of days from referral to DIH until the final diagnosis was made (hospital interval), and finally, based on these 2 intervals, the total number of days from first consultation in primary care until the diagnosis was calculated (diagnostic interval).

Date of first consultation was set to the date where the patient consulted a primary care provider and presented symptoms and signs that most probably could be ascribed to CRC.

Date of referral was the date where a primary care provider sent a written or verbal referral to DIH surgical department for further diagnostic actions and management of the patient. The date were collected from the primary care IT system.

Date of final diagnosis was the date where the pathology report was obtained of the first histological or cytological sample confirming CRC.

### Statistics

STATA was used for data analysis. Time intervals were found to be right skewed and therefore a non-parametric test was used. Descriptive data were presented as frequencies, fractions, medians and interquartile ranges and, where appropriate, the Mann–Whitney test was applied. P-values below 0.05 were considered significant.

## Results

**Figure 1.**
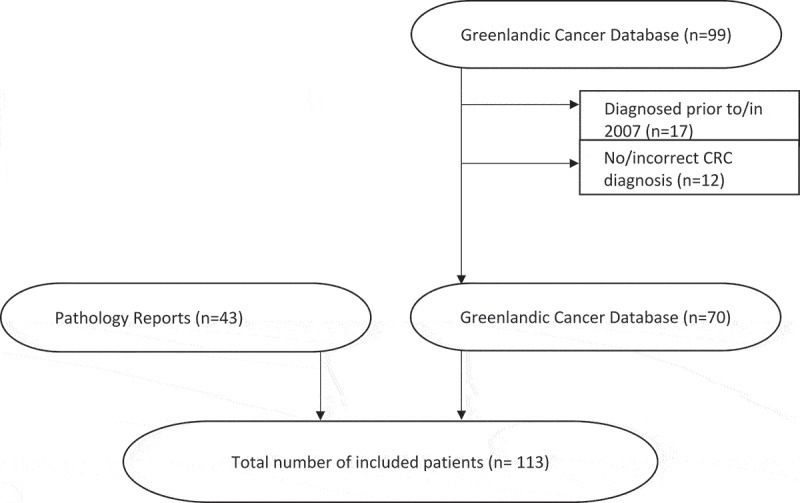
Final study sample of patients with CRC diagnosed from 2008–2011. Seventy patients were identified through the Greenlandic cancer database and an additional 43 cases were identified from pathology reports.

A total of 113 patients were included in this study (Figure 1); 83 patients had cancer in the colon (73%) and 30 had cancer in the rectum (27%). The majority of the patients (75%) came from “the Coast”, and the rest (25%) from Nuuk (Table 1).

**Table 1.** Descriptive statistics of the population with CRC from 2008 to 2011 in Greenland.

**Average age at diagnosis (years)**	61.2
	**No. (%)**
**Total number of colorectal cancer**	113
Colon cancer	83 (73)
Rectal cancer	30 (27)
**Male**	64 (57)
**Female**	49 (43)
**Co-morbidity**	
Yes	25 (22)
No	65 (58)
Not reported	23 (20)
**Geographic location**	
Nuuk	28 (25)
Outside Nuuk	85 (75)

**Figure 2.**
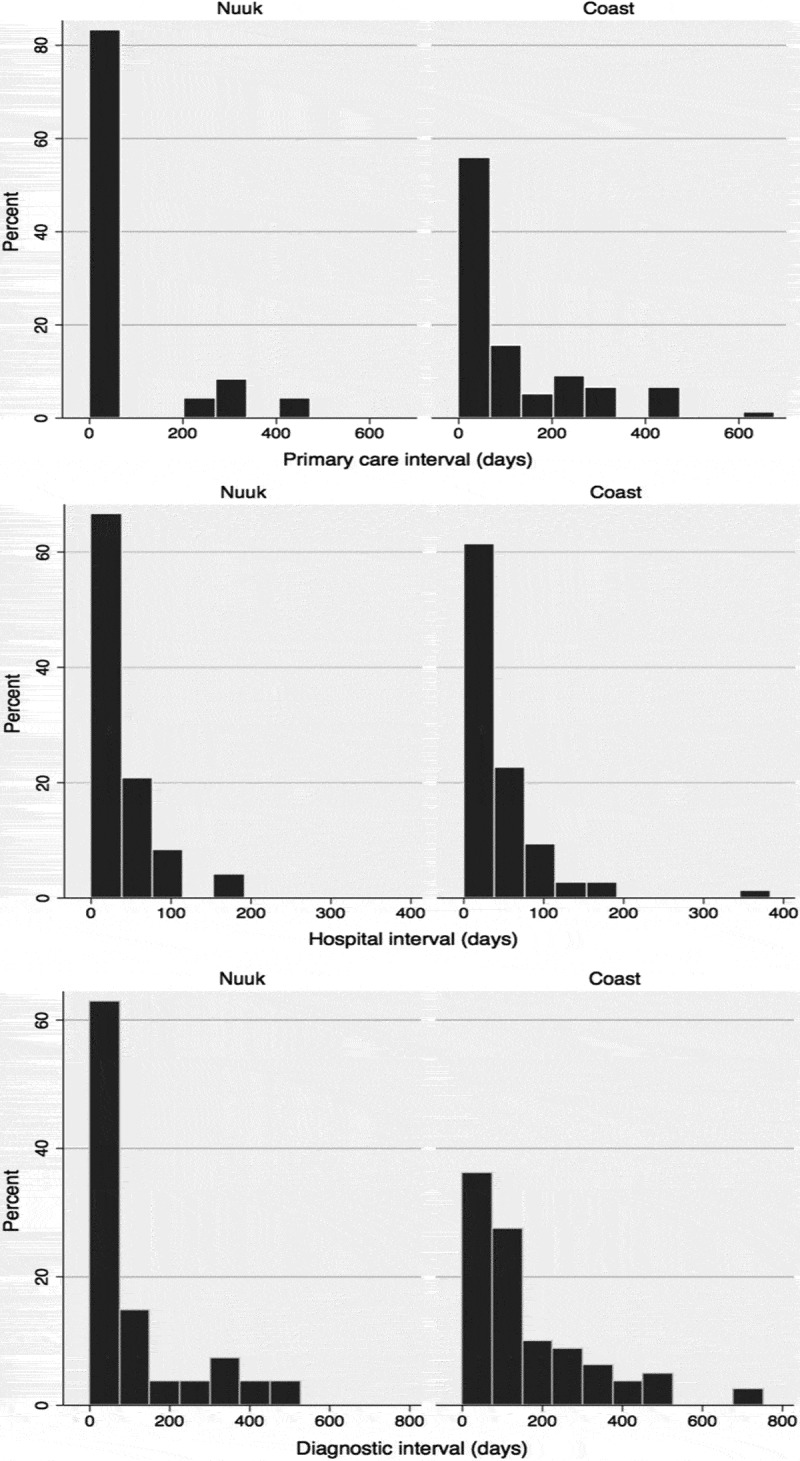
Percentages of patients from respectively Nuuk and “The Coast” and duration of the different diagnostic intervals.

### Symptoms

Of the patients, 42 patients (37%) had abdominal pain or pain at defecation as their first symptom of CRC. Thirty-six (32%) presented themselves with blood in the stools and 34 (30%) with changes in bowel habits. Thirty-four (30%) patients described general malaise or signs of anaemia as the first sign of CRC. Almost half of the patients with general malaise also had anaemia. A total of 13% of the patients presented with unintended weight loss, 2% with a tumour mass in the abdomen and 2% had no records describing their symptoms (Table 2).

**Table 2.** First symptoms of CRC, diagnosis of referral and mode of referral.

**First symptom of CRC**	**No. (%)**
Abdominal pain /pain at defecation	42 (37)
Blood in the stools	36 (32)
Changes in bowel habits	34 (30)
General malaise and symptoms of anaemia	34 (30)
Unintended weight loss	15 (13)
Abdominal tumour mass	2 (2)
Not reported	2 (2)
**Diagnosis of referral (suspicion)**	
Colorectal cancer	49 (43)
Gastrointestinal bleeding/anaemia	22 (20)
Ileus/subileus	12 (11)
Abdominal tumour (not specified)	8 (7)
Benign gastrointestinal disease	7 (6)
Other diagnosis (LUTS, heart failure, lung cancer)	6 (5)
Not reported	9 (8)
**Mode of referral**	
Acute	17 (15)
Subacute (first possible flight connection)	18 (16)
Ordinary	78 (69)

Sixty-two patients (55%) had only 1 symptom of CRC registered at their first contact with the health care facility, while 49 (43%) had more symptoms registered (i.e. both blood in the stools and stomach pain).

The majority of patients were referred to further examinations at DIH under the suspicion of CRC (43%) or a non-specified tumour in the abdomen (7%). Twenty percent were referred due to gastrointestinal bleeding and thus, to some extent, suspicion of malignancy. Eleven percent of the patients were admitted with signs of ileus. Finally, some patients were referred with a suspected benign gastrointestinal disease (6%) and some with symptoms of prostate hypertrophy, congestive heart failure and lung cancer (5%). For 8% of the patients, no referral diagnosis could be retrieved. Seventeen patients (15%) were admitted/referred acutely to DIH, mainly due to ileus or gastro-intestinal bleeding, 18 (16%) were admitted sub-acutely by first possible flight connection and the rest, 78 patients (69%), were referred through standard procedures (Table 2).

### Medical history

In 84% of all medical files notes the primary provider addressed changes in bowel habits, and in 78% notes regarding blood in the stools were found. For 78% of the patients, the provider addressed the question of stomach pain/pain at defecation and for 78% general malaise/symptoms of anaemia was addressed. Finally, unintended weight loss was addressed for 55% of the patients.

### Objective examinations and haemoglobin measurement prior to referral

For 78% of the patients, abdominal examination had been carried out and a rectal examination was performed for 65%. Thirty percent of the women had a gynaecological examination performed. Twenty-two percent had a proctoscopy made, and of these 6 patients were not referred afterwards despite of symptoms of CRC.

Fifty-one percent had the haemoglobin level measured, and 67% of these had anaemia (Table 3).

**Table 3.** The frequency of which medical history, objective examination and haemoglobin measures were described in the medical records.

**Medical history**	**No. (%)**
Changes in bowel habits	95 (84)
Blood in the stools	89 (78)
Abdominal pain /pain at defecation	88 (78)
General malaise	88 (78)
Unintended weight loss	62 (55)
**Objective investigations**	
Abdominal palpation	88 (78)
Rectal examination	73 (65)
Gynaecological examination	15/50 (30)
Proctoscopy	25 (22)
**Haemoglobin measures**	58 (51)
Fraction with anaemia	39 (67)
Fraction without anaemia	19 (33)

### Comparison between time intervals for Nuuk and “the Coast” (Table 4 and Figure 2)

Patients from Nuuk had a median primary care interval of 4 days and a median hospital interval of 23 days. Median diagnostic interval was 55 days.

**Table 4.** Primary care interval, hospital interval and diagnostic interval.

	**Quartiles**				
	Min	0.25	Median	0.75	Max
**Nuuk**					
Primary care interval (n=24)	0	0	4		415
Hospital interval (n=24)	4	11	23	60	166
Diagnostic interval (n=27)	4	17	55*	126	466
**Outside Nuuk**					
Primary care interval (n=77)	0	5	55*	171	677
Hospital interval (n=75)	0	10	24	66	346
Diagnostic interval (n=80)	0	52	95*	246	753
**Men**					
Primary care interval (n=57)	0	1	15	93	441
Hospital interval (n=56)	0	8	23	65	346
Diagnostic interval (n=60)	2	32	80	179	753
**Women**					
Primary care interval (n=44)	0	6	70	210	677
Hospital interval (n=43)	0	13	32	53	166
Diagnostic interval (n=47)	0	56	98	251	752

*Indicates significant difference between patients from Nuuk compared with patients living outside Nuuk.

Patients from “the Coast” had a median primary care interval of 55 days and a median hospital interval of 24 days. The median diagnostic interval was 95 days.

Patients from “the Coast” had a significantly longer primary care interval and also diagnostic interval compared with patients from Nuuk (p=0.01 and p=0.04). There was no significant difference in hospital interval between the 2 groups (p=0.86).

### Comparison between men and women

The abovementioned time intervals were assessed for both men and women (Table 4).

The median primary care interval was 15 days for men vs. 70 days for women; however, this difference was non-significant (p=0.06). We found a similar non-significant difference regarding the median diagnostic interval with men passing 80 days and women 98 days (p=0.10). No significant difference was found regarding the median hospital interval (p=0.74).

## Discussion

We assessed the symptomatology of Greenlandic patients with CRC and estimated the primary care interval and hospital interval for patients from Nuuk vs. the rest of Greenland as well as the diagnostic interval. To our knowledge this has not been studied in an Arctic population before. Overall the Greenlandic patients with CRC presented with classic symptomatology, and the 3 most frequent symptoms were abdominal pain, blood in the stools or changing bowel habits [7].

Median diagnostic interval was significantly different with, respectively, 55 days for patients from Nuuk and 95 days for patients from “the Coast”. A previous study in a Spanish population found, more or less in accordance with our results, a median diagnostic interval of 66 days [11], while an American study found this time interval to be of 3 weeks and thus substantially shorter [7]. A Swedish study found a median diagnostic interval of 33 days from patients with colon cancer and 24 days for patients with rectal cancer [12]. These studies are carried out in different settings and thus not directly comparable.

Early detection of CRC can reduce mortality [10]; however, it is not yet clear whether diagnostic and therapeutic intervals increase mortality for patients with CRC [8], but a Danish study indicates an association between longer diagnostic intervals and mortality [9]. Even if this association is not present, the national legislation on health care delivery states that all patients independent of their place of living should be given an equivalent service. It is a serious challenge to the Greenlandic health care system in general, as well as to the success of the national cancer plan, that the population seems to be offered a more efficient and timely primary care treatment in Nuuk compared with the rest of the country in the case of CRC.

It is likely that the distances in Greenland play a crucial role. A study from Tasmania found that practitioners in rural areas were more reluctant to refer patients with rectal bleeding to a specialist [13]. A population-based study from Canada found that living in rural compared with urban communities was a strong predictor of lower referral rates for patients; this study, however, did not explicitly describe referrals related to CRC [14].

The continuity of care in Greenland is also challenged due to shifting and temporary health care staff in many clinics at “the Coast”. A systematic review from 2010 found provider continuity to be associated with better patient outcome and patient satisfaction [15], and from the review of Vega et al. some evidence suggests that lack of continuity of care is associated with longer primary care interval [16].

In a review from 2008 2 main reasons for longer primary care intervals were identified: initial misdiagnosis and insufficient examination [17]. Diagnosing CRC is difficult, due to the often vague and unspecific nature of the symptoms [18], and a previous study by Tørring et al. showed that patients with CRC presenting with vague symptoms experienced long diagnostic intervals compared with patients with alarm symptoms [9]. We were not able to assess if patients from Nuuk presented with more “alarm” symptoms and, by consequence, had a shorter primary care interval.

Our data did, however, indicate that the questioning and objective investigations before referral of the patients were not being performed in a sufficiently thorough way and according to guidelines in more patients. No major differences were seen between Nuuk and “the Coast” in this matter. The majority of the patients were asked about changes in bowel habits, but almost 25% of the patients had no questioning recorded regarding blood in the stools, abdominal pain and general malaise/anaemia. Unintended weight loss is a symptom of many different cancer diseases and many patients with this symptom await several months before seeking medical assistance [7]. In our study this topic was addressed for only 55% of the patients. Previous studies also found that many patients, where CRC was later diagnosed, were not properly examined [11,19,20]. A Spanish study found that only around one-third of the patients had abdominal and rectal examination performed [11]. Patients from our study were examined more extensively than this, but only, respectively, 78% and 65% had an abdominal examination and a rectal examination performed. Gynaecological examinations were made for less than a third of all females. More than one-fifth of our patients had a proctoscopy made, a procedure which is regarded as obsolete due to insufficient diagnostic value compared with other endoscopic procedures [21]. Six patients had a “false negative” proctoscopy performed, and it probably contributed to a delay in the diagnostic process. Measurement of haemoglobin was only performed for half of the patients despite easy access to the test in most places. Ideally, the type of anaemia should be defined with further blood samples (s-ferritin) as especially IDA may be caused by CRC, and thus this marker may serve as a useful adjunct to the overall assessment of the patient [22]. IDA is present for 11–57% of patients with CRC; in particular, when the tumour is located to the right side of the colon as much as 65–80% have IDA [23].

We found that women had a longer primary care interval than men, even though this difference was non-significant (p=0.06). However, we could not find other studies with similar findings; on the contrary, 2 reviews, from 2015 and 2008, failed to find such gender differences [16,17]. In our investigation women often presented with symptoms consistent with a urinary tract infection, menopause or pelvic inflammatory disease, and thus it is possible that the diagnosis of CRC could be masked by such benign causes.

Regarding socioeconomic factors, education and income may also play a role in the differences in primary care interval. Overall, citizens in Nuuk have higher socioeconomic status compared with those from “the Coast” [24], and a review by Mitchell et al. from 2008 [17] demonstrated an association between lower socioeconomic status and longer primary care interval for patients with CRC. The reason for this is not clear and is probably complex, but it is reasonable to assume that educated patients are better informed regarding potential diseases and may be more explicit in their wish to be referred.

Screening for CRC is under consideration in Greenland, since it has been shown that individuals participating in CRC screening have a lower cancer stage at time of diagnosis, they can have pre-cancerous lesions removed and overall they have a reduced mortality [25]. Such an initiative could possibly help to reduce the differences in primary care intervals for those participating due to the nature of screening as a standardised examination for the whole population independent of geography. Also a general increased focus on CRC in the population and amongst health care providers could be expected. The evidence for CRC screening is based on studies using the traditional guiac-based haemocult test (G-FOBT), but recently the immunochemical faecal occult blood test (I-FOBT/FIT) has been recommended in many countries including Denmark [26], since this test has better detection rates for advanced adenomas and cancer. Also, I-FOBT is specific for human (not meat) haemoglobin in faeces, and this therefore test has no dietary or drug restrictions [27], which could be crucial in Greenland where the diet is often meat based. However, the cost effectiveness should be carefully evaluated. The Department of Health has mainly explored 2 models of screening: the first is the Danish model that includes a FIT test followed by a colonoscopy if the test is positive and screening with a new FIT test every second year if negative. The other is the “Alaskan tribal model” that offers colonoscopy and follow-up after 10 years if negative. Both include a follow-up of adenomas, and the economic burden is not due to the primary tests but in the follow-up since colonoscopy is today only performed in Nuuk. Even if colonoscopy is offered also at the regional hospitals, the costs of implementing the Alaskan model in Greenland would be about 3% [28] of the national health care costs, which is around 1.3 billion DKK. The costs of implementing the Danish screening model is less, but has not yet been fully explored, and more studies are warranted before screening can be implemented [26].

Individuals from urban areas and with higher socioeconomic status have higher screening rates [29,30] leading to better survival from CRC, and thus screening for CRC may increase inequalities in Greenland. If a screening programme is implemented actions should be taken to avoid such inequalities. Essink-Bot et al. suggest, for example, that the invitation to CRC screening could be adjusted to different socioeconomic classes [31].

## Limitations

Apart from the retrospective nature of the study, further limitations should be addressed: missing data may have biased some of our conclusions. We are not able to state more clearly if this is due to a flaw of documentation or if the patients have bypassed the GP/primary care setting. Missing data could be due to an immediate referral where the GP has not stated this in the medical record. In such cases our data may overestimate the primary care interval on “the Coast”. Furthermore, to be able to identify the relevant symptom or symptoms of CRC, a subjective estimation had to be made for every patient; for example, a patient with diarrhoea and abdominal pain 1 year before the diagnosis of CRC could have gastroenteritis, and not necessarily symptoms of CRC. Since we knew, in advance, that the patients had cancer, the researchers may have been prone to ascribe too many symptoms to the cancer. However, this was mainly done by the same physician, with indepth knowledge of CRC, making this registration as standardised as possible. Unfortunately, no validation of the data was made, making it more prone to wrong assessment and errors.

We were not able to assess the question of co-morbidity, income, education and ethnicity, factors that may have influenced the differences in diagnostic intervals. However, this does not change the fact that the difference is present, regardless of the underlying reason.

Another limitation is the fact that we could only register what was actually described in the medical files (recording bias). It is likely that more patients than the numbers given above were questioned and examined more extensively, and that this was simply not documented in the medical reports. We had no chance to investigate this hypothesis. If this theory is partly correct, there is a flaw in documentation after many consultations.

Unfortunately, we did not keep records about family disposition to CRC; however, our clear impression is that such questions are rarely asked.

Finally, this study is based on data from 2008 to 2011. Since then, the number of patients that are examined with endoscopy (especially colonoscopy) has increased in Greenland, and hopefully this has contributed to a better detection rate of CRC. More high-quality research is needed to elucidate these questions; for example, would patients with CRC be detected at a lower stage today?

## Conclusion

Primary care interval and diagnostic interval are significantly longer for patients with CRC living outside Nuuk, where the national hospital is situated, with no difference, however, regarding hospital interval. Women seem to wait longer before referral compared with men, and thus the practitioner should pay particular attention to female patients. The most important reason for longer diagnostic intervals for patients living outside Nuuk is not clear. Logistical challenges, lack of continuity of care and easy access to colonoscopy is probably a substantial part of the explanation. However, actions should be taken, especially outside Nuuk, to promote guidelines regarding detection of CRC, since the primary questioning and objective investigations are not sufficiently standardised or fully performed. This may lead to poorer diagnostics and treatment. Proctoscopy should not be performed, and haemoglobin should be measured more often for patients with symptomatology compatible with CRC. A national screening programme could improve detection rates of CRC and reduce CRC-related mortality; however, cost effectiveness is not yet fully investigated. These abovementioned initiatives could ensure a correct and expedited referral to investigation and treatment for patients with CRC, especially for inhabitants living outside the capital.
